# Complete genome sequences of microbacterium phages Tedro and BAjuniper

**DOI:** 10.1128/MRA.00793-23

**Published:** 2023-10-31

**Authors:** Grace M. Anderson, Olivia E. Anderson, Clayton A. Bosma, Erin E. Brouwer, Kleyton M. DeGroot, Owen C. Hede, Daiki Jonouchi, Dylan J. Kirkeby, Jayron T. Klinghagen, Kate J. Kralik, Julia S. Kutz, Olivia F. Lott, Erika J. McKenney, Victoria L. Pavik, Cayli H. Penner, Garrett Raymon, Leah B. Rozeboom, Jett L. Skrien, Jessica K. Slight, Maegan J. Stokes, Jonas D. Tiensvold, Kyra J. Wajer, Alaena L. Trevino, Byron Noordewier, Sara S. Tolsma

**Affiliations:** 1 Department of Biology, Northwestern College, Orange City, Iowa, USA; Department of Biology, Queens College, New York, USA

**Keywords:** bacteriophage, microbacterium phage

## Abstract

We purified two novel bacteriophages from soil collected in Sioux County, Iowa: BAjuniper and Tedro. These bacteriophages were isolated from the host, *Microbacterium foliorum*. BAjuniper was assigned to cluster EB, and Tedro was assigned to cluster EF. Both phages display genomes typical of other phages in their clusters.

## ANNOUNCEMENT

Scientists estimate that there are 10^31^ viral particles on our planet (1). Today, GenBank lists only about 12,000 viruses whose complete genomes have been sequenced and annotated, fewer than 5,000 of which are bacteriophages. The prokaryotic virosphere includes remarkable diversity, but we have only begun to scratch the surface of our understanding of that diversity (2). Here, we report the characteristics of two newly isolated actinobacteriophages, BAjuniper and Tedro, to contribute to our understanding of phage diversity.

**Fig 1:**
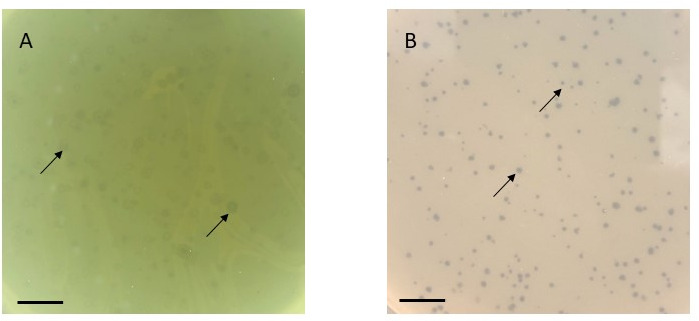
Plaque characteristics of phages BAjuniper and Tedro after 36 (BAjuiper) and 48 (Tedro) hours at 30°C. (A) BAjuniper produced turbid plaques of variable shape and size with a mean diameter of 4.6 mm ± 2.1 mm (*n* = 25). (B) Tedro produced clear, round plaques with a mean diameter of 1.0 mm ± 0.3 mm (*n* = 25). Image analysis was performed with ImageJ (4). Scale bars = 10 mm. Arrows indicate typical plaques for each phage.

BAjuniper and Tedro were isolated from soil samples 2–4 inches deep collected in Northwest Iowa using standard isolation methods (3) (Table 1). Briefly, we washed soil samples with peptone-yeast extract-calcuim (PYCa) liquid medium supplemented with 0.1% dextrose. We isolated and purified phages in the filtered wash (0.2 µm pore size, cellulose acetate; Corning, Glendale, AZ, USA) with at least three rounds of plating in PYCa top agar overlays with *Microbacterium foliorum* NRRL B-24224. BAjuniper formed turbid plaques with variable shape, and Tedro formed round, clear plaques after 24–48 hours at 30°C (Fig. 1).

**TABLE 1:** Isolation details, sequencing details, and genomic characteristics of BAjuniper and Tedro

Phage names	Soil sample GPS^*a*^ coordinates 43.25 N, 96.321 W	Approximate shotgun coverage	Genome size (bp)	Genome ends	G + C content (%)	# of protein-coding genes	# of tRNA genes	Cluster assignment
BAjuniper	43.15000 N 96.19156 W	516	41,985	3′ sticky overhang (5′-ACTCCCGGCA-3′)	68.8	65	3	EB
Tedro	43.00681 N 96.48255 W	311	56,197	Circularly permuted	63.7	83	0	EF

^*a*^ GPS, Global Positioning System.

We isolated phage DNA from high-titer lysates using the Wizard DNA Clean-Up System (Promega) and concentrated it with the DNA Clean and Concentrator Kit (ZYMO Research). We pooled the DNA samples and prepared them for sequencing on an Illumina MiSeq sequencer using the NEB Ultra-FS kit. Untrimmed single-end reads (average length of 165 bases) were assembled using Newbler (v2.9) and checked for completeness using Consed (v29.0), using default parameters (5, 6) (Table 1). The phage DNA sequences from the pooled sample were substantially different, enabling the assembly of two distinct genomes (5). BAjuniper was assigned to cluster EB and Tedro to cluster EF based on a gene-content similarity (GCS) of 35% or higher to sequenced bacteriophage genomes in the Actinobacteriophage database, phageDB [(7), accessed 15 August 2023] using the phagesDB GCS tool and criteria that have been previously described (8). Details of sequencing, genome characteristics, and cluster assignments are reported in Table 1.

We annotated both genomes using DNA Master v5.23.2 (cobamide2.bio.pitt.edu) with embedded Glimmer v3.02 and Genemark v2.5, PECAAN (https://discover.kbrinsgd.org/), Phamerator, Starterator (http://phages.wustl.edu/starterator/), HHPRED, BLASTp, Aragorn v1.1, and tRNAscan-SE v2.0, all using default parameters (9 – 15). BAjuniper’s genome includes 65 protein-coding genes and 3 tRNA genes (tRNA^pro^, tRNA^asn^, and tRNA^gln^). We identified 83 protein-coding genes in Tedro’s genome and no genes encoding tRNAs.

We identified programmed translational frameshifts in the putative tail assembly chaperone genes for BAjuniper and Tedro (gp13/14 and gp27/28) (16). Both phage genomes include genes that encode putative large and small terminase genes (gp2 and gp3 in BAjuniper, gp4 and gp11 in Tedro) (17). Although we were not able to identify integrase or excise genes in either genome, which is consistent with the predicted lytic lifecycle for phages in these clusters, we note the presence of a putative recombination directionality factor (gp32) gene and putative Holliday junction resolvase (gp36) genes in BAjuniper and other cluster EB phages. These gene products have been implicated in the establishment of lysogeny for some phages, though they can also play other non-lysogeny-related functions (18 – 20). Both phage genomes encode proteins (gp53 in Tedro and gp30, gp31, gp56, and gp58 in BAjuniper) involved in DNA metabolism (21, 22).

## Data Availability

The complete genome sequences of phages BAjuniper and Tedro are available in GenBank (accession nos. OQ938582 and OQ938591, respectively). The raw sequencing reads are available in the NCBI SRA under accession no. SRX20630256 and SRX20630259, respectively.
